# Supplementary material for: Mammalian Brain Ca2+ Channel Activity Transplanted into Xenopus laevis Oocytes
Source: Membranes (Basel). 2022 May 2;12(5):496. doi: 10.3390/membranes12050496 (PMC9146698; doi:10.3390/membranes12050496)
Supplement: Supplementary file 1 [file membranes-12-00496-s001.zip › membranes-1638647-supplementary.pdf]

A

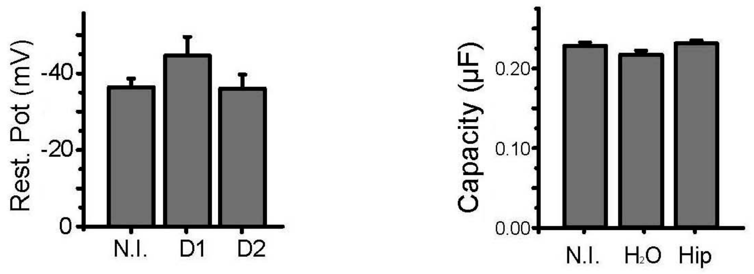

B

Subunit	MW (kDa) human	MW (kDa) mice
CaVβ1a	57.8	CaVβ1.4: 57.8
CaVβ1b	65.7	CaVβ1.1: 65.5
CaVβ1c	53.2	CaVβ1.3: 52.9
CaVβ1d	---	CaVβ1.2: 74.1
CaVβ2a	68.1	CaVβ2.1: 73.1
CaVβ2b	68.2	CaVβ2.2: 68.2
CaVβ2c	70.8	CaVβ2.3: 68.8
CaVβ2d	73.6	CaVβ2.4: 64.7
CaVβ3a	54.5	CaVβ3.a: 54.6
CaVβ3b	50.1	----
CaVβ3c	53.1	----
CaVβ3d	54.3	----
CaVβ4a	58.2	CaVβ4.1: 57.9
CaVβ4b	54.7	CaVβ4.2: 54.6
CaVβ4c	56.5	CaVβ4.3: 53.1
CaVβ4d	51.3	----

Supplementary Figure S1. A. *Passive properties of membrane-transplanted oocytes.* Averaged membrane resting potential (left), and oocyte membrane capacitance (right) of oocytes non injected (NI), injected with water (H2O) or injected with hippocampal membrane preparation (Hip, 7 mg/ml) one (D1) or two days (D2) after membrane injection. B. Molecular weight of different Cavβ subunits. *(Source: unitprot.org).*
